# Alcohol and E-Cigarettes Activate P2X7R in Lung Epithelial Cells, Stimulating Release of Plasminogen Activator in Extracellular Vesicles, Injuring Blood-Brain Barrier

**DOI:** 10.3390/cells15191750

**Published:** 2026-09-25

**Authors:** Naveen Mekala, Namdev Togre, Sunil Kurian, Slava Rom, Uma Sriram, Yuri Persidsky

**Affiliations:** 1 Department of Pathology and Laboratory Medicine, Lewis Katz School of Medicine, Temple University, Philadelphia, PA 19140, USA; nst5227@psu.edu (N.T.); slava.rom@temple.edu (S.R.); uma.sriram@temple.edu (U.S.); 2 Scripps Clinic and Biorepository, Scripps Center for Organ and Cell Transplantation Research, La Jolla, CA 92037, USA; smkurian@scripps.edu

**Keywords:** Ethanol, e-cigarette vape, P2X7 receptor, urokinase-type plasminogen activator, lung–brain cross talk

## Abstract

**Highlights:**

**What are the main findings?**

- Alcohol and e-cigarette exposure activates a P2X7R–NLRP3 axis in lung epithelial cells, promoting release of uPA-enriched EVs that induce plasmin/MMP-9–TGF-β1 signaling and BBB dysfunction in brain endothelial cells.
- Pharmacological P2X7R inhibition reduces EV-associated uPA release and protects against BBB injury, while chronic ethanol exposure in vivo increases uPA/uPAR and MMP-2/MMP-9 and decreases SERPIN1/TIMP1 in brain microvessels.

**What are the implications of the main findings?**

- EV-associated uPA may represent a novel lung-to-brain signaling mediator linking alcohol and e-cigarette exposure to neurovascular dysfunction.
- The P2X7R–EV-uPA axis may provide a potential therapeutic target for preventing BBB disruption and downstream neurovascular injury associated with chronic alcohol and e-cigarette exposure.

**Abstract:**

Chronic alcohol and nicotine exposure can increase susceptibility to lung injury and promote the release of extracellular ATP (eATP). Elevated eATP activates the purinergic P2X7 receptor (P2X7R), a membrane ion channel that amplifies inflammatory signaling and promotes extracellular vesicle (EV) release. These EVs may serve as mediators of lung to systemic communications. Proteomic analysis of lung epithelial cell-derived EVs revealed enrichment of urokinase-type plasminogen activator (uPA), a key enzyme involved in extracellular matrix remodeling. Treatment of hPAEpiCs with ETH, acetaldehyde (ALD), and e-cigarette vapor, with or without nicotine, induced NLRP3 inflammasome activation, IL-1β secretion, and increased incorporation of uPA into EVs. Exposure of human brain microvascular endothelial cells (hBMVECs) to these EVs enhanced plasmin conversion and activated MMP-9 and TGF-β1, resulting in reduced transendothelial electrical resistance (TEER), indicating blood–brain barrier (BBB) dysfunction. Pharmacological inhibition of P2X7R using ‘A804598’ significantly reduced EV-associated uPA release and attenuated endothelial injury. In vivo, chronic ETH vapor exposure increased expression of uPA, uPAR, MMP-2, and MMP-9 while decreasing SERPIN1 and TIMP1 in murine brain microvessels, changes associated with BBB disruption and IgG leakage into brain parenchyma. P2X7R inhibition normalized these effects. Collectively, these findings identify EV-associated uPA as a mediator of lung-to-brain signaling and BBB injury following ETH and e-cigarette exposure.

## 1. Introduction

Alcohol consumption among young adults has remained a significant public health concern for decades, and the recent rise in e-cigarette (e-Cig) use has further intensified these concerns in the United States [1,2]. While the deleterious effects of alcohol on organs such as the liver, brain, and gastrointestinal tract are well established, its impact on the respiratory system is increasingly recognized [3]. Alcohol consumption elevates the risk of acute respiratory distress syndrome (ARDS) by compromising alveolar epithelial barrier integrity and promoting fluid accumulation in the alveolar space, thereby impairing gas exchange and leading to respiratory failure [4].

E-cigarettes, typically containing 1–2.4% nicotine, are often marketed as safer alternatives to conventional cigarettes [5]. However, to remain competitive, manufacturers have increased nicotine concentrations in e-liquids to ≥4.3% [6,7]. Inhalation of high concentrations of nicotine, along with volatile organic compounds and metal particles generated during heating, can damage alveolar structures critical for gas exchange [8]. At the molecular level, cigarette smoke has been shown to activate purinergic receptors (P2X) and transient receptor potential (TRP) channels, promoting reactive oxygen species (ROS) production in the lungs [9]. Activation of the P2X7 receptor (P2X7R) further contributes to inflammatory cytokine release and cellular injury, exacerbating lung damage [10].

Our previous studies demonstrated that ethanol (ETH) and e-Cig exposure (1.8% nicotine) activate P2X7R and impair mitochondrial function in human pulmonary alveolar epithelial cells (hPAEpiCs) [11]. We also showed that these exposures enhance extracellular vesicle (EV) release from hPAEpiCs. These EVs carry damage-associated molecular patterns (DAMPs), including ATP and mitochondrial DNA (mtDNA), which induce calcium (Ca^2+^) accumulation in human brain microvascular endothelial cells (hBMVECs). In addition to DAMPs, EV protein cargo plays a critical role in intercellular signaling, regulating processes such as cell proliferation, extracellular matrix (ECM) remodeling, and cell death [11,12]. In the present study, we employed liquid chromatography–mass spectrometry (LC–MS) to characterize the protein cargo of hPAEpiC-derived EVs.

Urokinase-type plasminogen activator (uPA) is a serine protease involved in fibrinolysis and ECM remodeling during inflammation and cancer progression [12,13]. Binding of uPA to its receptor (uPAR) converts plasminogen to plasmin, which in turn activates matrix metalloproteinases (MMP2, MMP9, and MMP13) [14]. Plasmin also converts latent TGF-β1 into its active form, promoting ECM remodeling and cell migration [15]. Beyond pathological conditions, uPA overexpression has been reported in models of substance abuse, including ethanol and methamphetamine exposure, where it contributes to blood–brain barrier (BBB) disruption [16,17]. However, the underlying molecular mechanisms remain poorly understood.

Activation of uPA–uPAR signaling in the brain stimulates neurons, astrocytes, and microglia, engaging downstream pathways such as MAPK and NF-κB that drive inflammatory cytokine production [18,19,20]. In neurodegenerative diseases, including Alzheimer’s and Parkinson’s disease, this signaling axis enhances microglial activation and MMP release, contributing to progressive neuronal damage [21,22]. Emerging evidence indicates that EV-associated uPA–uPAR signaling may further amplify proteolytic and inflammatory responses within the neurovascular unit [23]. Given its responsiveness to cellular stress, uPA is increasingly recognized as both a biomarker and a potential therapeutic target [24,25].

Proteins encapsulated within EVs are protected from degradation, enhancing their stability and persistence in the extracellular environment [26]. EV-mediated delivery facilitates the transfer of these proteins to endothelial cells, including those of the BBB [27]. Notably, uPA packaged within EVs can remain functionally active and promote angiogenesis at sites of tissue repair [23]. Similarly, tissue-type plasminogen activator (tPA) has been shown to cross the BBB in models of cerebral ischemia, contributing to barrier disruption [28,29]. EV-mediated transport may further prolong plasminogen activity and amplify its pathological effects in the brain microenvironment [30,31].

In this study, we aimed to determine how ETH, ALD, and e-Cig vapor alter the inflammatory cargo and functional effects of hPAEpiC-derived EVs. Using LC-MS/MS, we identified EV-associated inflammatory and proteolytic proteins and investigated the role of P2X7R-NLRP3 signaling in uPA cargo packaging. We further assessed EV-mediated BBB dysfunction in hBMVECs by measuring TEER, active MMP-9, and TGF-β1, and confirmed P2X7R involvement using A804598 (A80). Finally, using a chronic intermittent ethanol (CIE) mouse model, we evaluated brain microvascular ECM remodeling and BBB damage through analysis of uPAR, MMPs, and TIMP1.

## 2. Materials and Methods

### 2.1. Reagents and Kits

Ethanol (ETH, 200 proof) procured from (Decon Laboratories Inc, Cat. No. 2716, King of Prussia, PA, USA), acetaldehyde (ALD, 99.5% pure) from (ACROS Organics, Cat. No. 402788, Geel, Belgium), and e-cigarette (e-Cig) liquids containing 1.8% and 0% nicotine were obtained from (Pure E-Liquids, Peterborough, PE11SB, UK), respectively. The P2X7 receptor inhibitor A804598 (hereafter referred to as A80; Cat. No. 4473), urokinase-type plasminogen activator (uPA) inhibitor BC11 hydrobromide (Cat. No. 4372), and NLRP3 inhibitor CRID3 sodium salt (Cat. No. 5479) were purchased from (Tocris Bioscience, Bristol, UK) and dissolved in DMSO (Sigma-Aldrich, Cat. No. D5879, St. Louis, MO, USA) or sterile water according to manufacturer instructions. Exosome isolation was performed using the ExoQuick-TC kit (SBI, Cat. No. EXOTC50A, Palo Alto, CA, USA). Human Active MMP-9 and IL-1β ELISA kits were obtained from (R&D Systems, Cat. No. F9M00 and DLB50, Minneapolis, MN, USA), and human plasmin ELISA kit was obtained from (LS Bio, Cat. No. LS-F2748, Newark, CA, USA).

### 2.2. Cell Culture and Treatments

Human pulmonary alveolar epithelial cells (hPAEpiCs; Accegen, Cat. No. ABC-TC3770, Fairfield, CT, USA) were maintained in hPAEpiC basal medium (Accegen, Cat. No. ABM-TM3770) supplemented with insulin-transferrin-selenium, epidermal growth factor, hydrocortisone, and 5% exosome-depleted fetal bovine serum (FBS; Gibco, Cat. No. A2720801, Waltham, MA, USA). Human brain microvascular endothelial cells (hBMVECs, D3 line) were kindly provided by Dr. Pierre-Olivier Couraud (Institut Cochin, INSERM U1016, CNRS UMR 8104, Université Paris Descartes, Paris, France) [32] and cultured in EBM-2 basal medium (Lonza, Cat. No. CC-3156, Walkersville, MD, USA) supplemented with EGM-2 SingleQuots (Lonza, Cat. No. CC-4176 Walkersville, MD, USA). Cells were cultured in appropriate vessels (96-well, 6-well, 100-mm plates, or T-75/T-150 flasks) pre-coated with bovine collagen (CELL Applications Inc., Cat. No. 123-100, San Diego, CA, USA).

Confluent hPAEpiCs were pretreated with or without A80 (10 μM) for 1 h, followed by overnight stimulation with ETH (100 mM), ALD (100 μM), or e-Cig vapor-conditioned media (1.75 μg/mL, 1.8% or 0% nicotine), with or without A80. A80-treated cells served as P2X7 receptor antagonist controls. To investigate lung–brain crosstalk, hBMVECs were stimulated with hPAEpiC-derived EVs. Unless stated otherwise, all experimental endpoints were assessed after overnight stimulation. Concentrations of ETH, ALD, e-Cig, and inhibitors were based on prior dose-response studies [11].

### 2.3. Extracellular Vesicle (EV) Isolation

EVs were isolated from hPAEpiC culture supernatants using ExoQuick-TC according to the manufacturer’s protocol. Briefly, culture supernatants were centrifuged at 3500 rpm for 15 min to remove cell debris and apoptotic bodies, filtered through a 0.45 μm membrane, and incubated with ExoQuick-TC reagent (1 mL per 5 mL supernatant) overnight at 4 °C. Precipitated EVs were pelleted by centrifugation at 3500 rpm for 15 min at 4 °C and resuspended in 100 μL sterile PBS.

### 2.4. Nanoparticle Tracking Analysis (NTA)

EV concentration and size distribution were determined using a NanoSight NS300 system (Malvern Panalytical, Malvern, UK) equipped with a 488 nm laser. Samples were diluted 1:1000 in particle-free MilliQ water and injected into the chamber using a 1 mL BD slip-tip syringe. Three 30 s videos per sample were recorded under continuous flow. These videos record and track the path of unlabeled particles/EVs acting as point scatterers, undergoing Brownian motion in the chamber using a laser beam [33] and analyzed with NTA 3.3.104 software. The system was calibrated with 100 nm latex beads (Malvern, Cat. No. NTA4088) prior to measurements. Representative EV particle sizes and numbers can be found in the Supplementary Files (Table S1).

### 2.5. Liquid Chromatography-Tandem Mass Spectrometry (LC-MS/MS)

We sent culture supernatants to Systems Biosciences (SBI, Newark, CA, USA), for LC-MS/MS analysis, which was performed using the following protocol.

#### LC-MS/MS Proteomics

EV protein samples were analyzed by LC-MS using Nano LC-MS/MS (Dionex Ultimate 3000 RLSCnano System, Thermo Fisher, Waltham, MA, USA) interfaced with Eclipse (Thermo Fisher). Samples were loaded on to a fused silica trap column Acclaim PepMap 100, 75 µm × 2 cm (Thermo Fisher). After washing for 5 min at 5 µL/min with 0.1% TFA, the trap column was brought in-line with an analytical column (Nanoease MZ peptide BEH C18, 130A, 1.7 µm, 75 µm × 250 mm, Waters, Milford, MA, USA) for LC-MS/MS. Peptides were fractionated at 300 nL/min using a segmented linear gradient of 4–15%B for 30 min (where A: 0.2% formic acid, and B: 0.16% formic acid, 80% acetonitrile), 15–25%B for 40 min, 25–50%B for 44 min, and 50–90%B for 11 min. Solution B then returns at 4% for 5 min for the next run.

A DIA (Data Independent Acquisition) workflow was used to analyze the eluted peptides. The MS scan range was set at 400–1200, resolution 12,000, with AGC set at 3E6 and ion time set as auto. An 8 m/z window was set to sequentially isolate (AGC 4E5 and ion time set at auto) and fragment the ions in C-trap with a relative collision energy of 30. The MS/MS analysis was recorded with a resolution of 30,000. Raw data were analyzed with a predicted library from the specified database and for a library-free search using DIA NN 1.8.1 [34] with the recommended settings. Consistent with earlier findings, expression of the protein of interest in EVs was further confirmed by Western blot analysis.

### 2.6. Brain Microvessel Isolation from CIE-Exposed Mice

Microvessels were isolated from C57BL/6 mice exposed to chronic intermittent ethanol (CIE) vapor (16 h/day, 4 days/week for 3 weeks) as previously described [35]. Mice were randomized into four groups: two chronic intermittent ethanol (CIE)-exposed groups and two control groups, with one CIE and one control group receiving the P2X7R antagonist Brilliant Blue G (BBG; 45 mg/kg). CIE mice were exposed to ethanol (ETH) vapors to achieve sustained systemic ethanol exposure with rapid intoxication while bypassing the gastrointestinal tract and minimizing liver metabolism [36]. For the in vivo studies, we used the water-soluble, FDA-approved P2X7R antagonist BBG in place of A80, which requires DMSO for solubilization. BBG was selected to improve aqueous solubility and facilitate systemic administration and bioavailability in vivo. Brains were harvested post-perfusion, and cerebellum, meninges, and large vessels were removed. Tissue was homogenized in ice-cold HBSS, centrifuged at 1000 *g* for 10 min, and resuspended in 17.5% dextran. The pellet was further processed through 100 μm and 40 μm strainers, and microvessels were collected, centrifuged, resuspended in Trizol, and stored at −80 °C.

### 2.7. Gene Expression (qPCR)

Total RNA was extracted from microvessels using TRIzol (Thermo Fisher, Cat. No. 15596026). cDNA synthesis was performed using the High-Capacity cDNA Reverse Transcription Kit (Thermo Fisher, Cat. No. 4368814) with 300 ng RNA. qPCR was carried out on a QuantStudio 3 Real-Time PCR System (Thermo Fisher).

### 2.8. Western Blotting

EV, cell and mouse brain lysate proteins were separated on 4–12% Tricine gels and transferred to nitrocellulose membranes. Membranes were blocked, incubated overnight with primary antibodies (1:1000), followed by IRDye secondary antibodies (1:5000), and imaged using the Odyssey system (LI-COR, Lincoln, NE, USA). Primary antibodies included uPA (GeneTex, Cat. No. GTX100465, Irvine, CA, USA), CD81 (56039S), CD9 (13174S), caspase-1 (3866T), caspase-8 (4790T), β-actin (4967S) are procured from (Cell Signaling, Danvers, MA, USA) NLRP3 (Abcam, ab263899, Waltham, MA, USA ), TGF-β1 (Thermo Fisher, MA1-21595), and Anti-Mouse IgG (H+L) (Jackson immunoresearch, 315-005-003, West Grove, PA, USA).

### 2.9. ELISAs

IL-1β, active MMP-9, and plasmin levels were quantified using commercial kits according to the manufacturers’ instructions. Briefly, lysates or culture supernatants were incubated with capture antibodies, washed, incubated with conjugates, and developed with substrate. Optical density was measured at 450 nm.

### 2.10. Transendothelial Electrical Resistance (TEER)

TEER was measured in hBMVEC monolayers to study the systemic response of hPAEpiC-EV cargo on barrier integrity. The TEER assay was performed using the 1600R electric cell-substrate impedance system (ECIS) (Applied Biophysics, Troy, NY, USA) as previously described [11,37]. The ECIS system provides real-time monitoring of changes in TEER. In brief, hBMVECs at 20,000 cells/well were plated on collagen type I-coated 96W20idf electrode arrays (Applied Biophysics). Cells were allowed to form monolayers until stable TEER values were reached. hBMVECs were pretreated with human fibrinogen native protein (0.5mg/mL) (Invitrogen, Cat. No. RP-43142, Carlbad, CA, USA), and TEER values were recorded continuously at 4000 Hz. The next day, hBMVECs were stimulated with lung epithelial EVs (1:500-for every cell 500 EVs), and we continued to record the TEER values for another 24 h. Actual data is presented in a graphical format using data from at least four replicates. A parallel set of experiments was conducted without fibrinogen pretreatment to evaluate the role of fibrinogen in barrier repair.

### 2.11. Statistical Analysis

Data are presented as mean ± SD. Normality was assessed with the Shapiro–Wilk test. For comparisons among multiple groups, one-way ANOVA with Dunnett’s post hoc test was applied. Statistical significance was set at *p* < 0.05 (GraphPad Prism v10).

## 3. Results

### 3.1. Proteomic Analysis Reveals Upregulation of uPA in hPAEpiC-Derived EVs Following ETH, ALD, and E-Cig Exposure

Human pulmonary alveolar epithelial cells (hPAEpiCs) were stimulated overnight with ETH-, ALD-, or e-Cig (1.8% and 0% nicotine)-conditioned media. Extracellular vesicles (EVs) were isolated from culture supernatants, and proteomic profiling was performed using LC-MS/MS. Raw protein counts were normalized to no-protein control (NPC) values, and EV protein levels were further normalized to non-treated (NT) hPAEpiCs. Comparative analysis across the four conditions, illustrated via Venn diagrams, identified both overlapping and uniquely expressed proteins (Figure 1A), with fold changes visualized in a heat map (Figure 1B).

Exposure to ETH, ALD, and e-Cig conditioned media significantly altered the expression of 397 proteins in hPAEpiC-EVs. Pretreatment with the P2X7 receptor (P2X7R) antagonist A80 largely restored these expression profiles. Notably, six proteins were consistently affected across all treatments, suggesting a common P2X7R-mediated pathway in the cellular stress response (Figure 1A). Among these, plasminogen activator urokinase (uPA) exhibited the most pronounced upregulation (2–4-fold), which was attenuated by A80 pretreatment, implicating uPA as a key mediator of this response (Figure 1B). Western blot analysis of hPAEpiC-EVs confirmed the LC-MS/MS findings (Figure 1D).

### 3.2. NLRP3 Inflammasome Activation by ETH, ALD, and E-Cig Drives uPA Overexpression in hPAEpiCs

Confluent hPAEpiCs were treated with ETH-, ALD-, or e-Cig-conditioned media, with or without A80. Cell lysates collected post-stimulation demonstrated significant upregulation of NLRP3 inflammasome components (3-fold), caspase-1 (2-fold), and caspase-8 (1.5-fold) (Figure 2A), consistent with enhanced IL-1β production [38,39]. Correspondingly, IL-1β levels in culture supernatants of treated hPAEpiCs ranged from 15–40 pg/mL, whereas IL-1β was undetectable in NT controls (Figure 2B). P2X7R inhibition markedly reduced NLRP3 activation and IL-1β secretion, highlighting its role in mediating the proinflammatory response.

To confirm the involvement of NLRP3 in uPA regulation, cells were pretreated with the NLRP3 inhibitor CRID3 (5 µM, 3 h) prior to ETH, ALD, or e-Cig exposure. Western blot analysis of EVs revealed that CRID3 pretreatment reduced the uPA content, supporting a possible mechanistic link between NLRP3 activation and uPA release (Figure 2C).

### 3.3. Chronic Ethanol Exposure In Vivo Upregulates ECM-Degrading Proteins in Brain Microvessels

Mice exposed to chronic ethanol vapor for three weeks exhibited elevated expression of uPA and uPAR in isolated brain microvessels (Figure 3A,B) [40], key mediators of plasminogen-dependent ECM degradation. This was accompanied by increases in MMP-9 (~100-fold) and MMP-2 (~20-fold) expression (Figure 3C,D) and decreased expression of SERPINE1 and TIMP1 (Figure 3E,F), indicating enhanced ECM proteolysis. Furthermore, Col1α1, a marker of fibroblast-mediated wound healing, was significantly upregulated (Figure 3G). P2X7R inhibition (BBG) normalized these gene expression changes, implicating P2X7R signaling in ethanol-induced blood–brain barrier (BBB) injury.

Our gene expression findings were further validated by Western blot analysis of brain lysates. Mice exposed to chronic ethanol showed the presence of total IgG in the brain compared with control animals (Figure S1), confirming BBB compromise. In addition, both IgG heavy and light chain levels were markedly increased following chronic alcohol exposure. Importantly, pharmacological inhibition of P2X7R with BBG significantly reduced the accumulation of total IgG, as well as heavy and light chains, in brain lysates, further supporting the likely role of P2X7R inhibition in preserving BBB integrity during alcohol exposure.

### 3.4. hPAEpiC-EV Cargo Compromises Brain Endothelial Barrier Integrity via Fibrinolysis

The functional impact of hPAEpiC-derived EVs on brain endothelial barrier integrity was assessed using transendothelial electrical resistance (TEER). hBMVEC monolayers without fibrinogen pretreatment stabilized TEER at ~600 Ω. Exposure to EVs from ETH-, ALD-, or e-Cig-treated hPAEpiCs (1:500) reduced TEER modestly (Figure 4A,B). In fibrinogen-pretreated monolayers (0.5 mg/mL), TEER increased to ~800 Ω; subsequent exposure to stimulated hPAEpiC-EVs decreased TEER by 20–30%, whereas EVs from NT cells had no effect (Figure 4C,D).

### 3.5. uPA Mediates EV-Induced Brain Endothelial Barrier Disruption

The role of uPA in ETH and e-Cig-induced endothelial barrier disruption was confirmed using BC11 hydrobromide, a selective uPA inhibitor [41]. hPAEpiCs were pretreated with 25 μM BC11 for 3 h prior to stimulation with ETH, ALD, or e-Cig vapor conditioned media. EVs were then isolated from the supernatant. A portion of these EVs was analyzed for uPA enrichment using Western blotting (Figure 5A). Against drugs of abuse, EVs released from BC11-pretreated cells showed markedly lower uPA levels.

Remaining EV fractions were used to evaluate their effects on hBMVEC barrier integrity by measuring TEER. Consistent with the previous experiment, treatment of hBMVEC monolayers with fibrinogen (0.5 mg/mL) increased endothelial resistance by 100 ohms. hPAEpiC-EVs generated against ETH, ALD, and e-Cig stimulation significantly decreased TEER values (10–20%), indicating barrier disruption (Figure 5B). Of note, hPAEpiC-EVs isolated from BC11 (uPA inhibitor)-pretreated cells showed no effect on electrical resistance values, suggesting that inhibition of uPA prevented barrier compromise. Uniquely, EV treatment from ETH- and e-Cig-exposed cells dropped the TEER values below those of the negative control (hBMVECs without fibrinogen treatment), further highlighting the important role of uPA-mediated proteolytic activity in compromising BBB with these insults. These observations provide evidence for a potential role of uPA generated from lung cells in damaging brain endothelial barrier integrity.

To connect the observed TEER decline with the expression and release of ECM-remodeling proteins, hBMVECs were stimulated with EVs derived from hPAEpiCs exposed to ETH, ALD, and e-Cig-conditioned media, and the levels of activated MMP9 and mature TGF-β1 were measured. EVs derived from substance-exposed hPAEpiCs significantly increased the secretion of activated MMP9 and mature TGF-β1 in hBMVECs (Figure S2). In contrast, EVs isolated from BC11-pretreated hPAEpiCs significantly attenuated MMP9 activation and TGF-β1 levels in hBMVECs, thereby mitigating the decline in TEER. Experimental data are available in the Supplementary File.

### 3.6. hPAEpiC-uPA Facilitates Plasmin Generation and ECM Remodeling in hBMVECs

uPA is a plasminogen activator known to activate downstream proteases, including MMP-2 and MMP-9 [42], which can proteolytically cleave latent TGF-β1 into its active form and promote ECM remodeling [43]. To confirm the role of hPAEpiC-derived uPA in mediating BBB disruption, we treated hBMVECs overnight with hPAEpiC-EVs (1:500). Active MMP-9 release was measured in the culture supernatant and active plasmin and TGF-β1 levels in cell lysates. EVs derived from control hPAEpiCs did not affect plasmin activation or MMP-9 secretion in hBMVECs. EVs from ETH- and ALD-treated hPAEpiCs enhanced plasmin activation (~50 pg/mL) and MMP-9 secretion (~2 ng/mL), while EVs from e-Cig–exposed cells strongly increased plasmin levels (50–100 pg/mL) (Figure 6A) and active MMP-9 release (0.5–1.5 ng/mL) (Figure 6B). Similarly, active TGF-β1 expression was significantly upregulated in hBMVEC lysates treated with EVs derived from ETH, ALD, and e-Cig stimulated cells (Figure 6D). Further, hPAEpiC-EVs pretreated with the P2X7R antagonist suppressed active MMP-9 release and attenuated the TGF-β1 expression, indicating a key role of P2X7R signaling in mediating these changes. However, EVs generated following 1.8% nicotine and A80 pretreatment failed to reduce TGF-β1 expression in hBMVECs, suggesting the involvement of an alternative signaling pathway in this response.

## 4. Discussion

According to a recent Gallup survey, traditional alcohol drinking in the U.S. has declined to its lowest level in 90 years, dropping from 60% to 54% [44]. Despite this, the practice of vaporizing and inhaling alcohol is gaining popularity among teenagers. Unlike traditional drinking, inhaled alcohol bypasses the digestive system and is rapidly absorbed into the bloodstream, leading to quicker intoxication [45]. Inhalation of alcohol vapors causes irritation in lung tissue, make it vulnerable to respiratory infections, and long-term exposure results in inflammation and injury [46]. Similarly, e-Cig vaping promotes the secretion of proinflammatory cytokines like IL-1β, IL-6, IL-8, and tumor necrosis factor alpha (TNF-α) in lung epithelial cells and throughout the upper airways [47]. Sustained inflammation driven by these cytokines can ultimately result in chronic lung injury [48]. In response to injury, lung cells/tissue release extracellular ATP (eATP) through regulated secretion or passive leakage from damaged cells [49]. Elevated eATP levels in the extracellular space act as ligands for purinergic receptor families such as P2X and P2Y, triggering and amplifying inflammatory responses within the same cells (autocrine signaling) and in neighboring cells (paracrine signaling) [50].

In our previous studies, we reported that ETH and e-Cig exposure promotes the P2X7R activation in hPAEpiCs, leading to elevated mitochondrial stress. Activation of P2X7R also promotes the opening of a large, non-selective cationic “macropore” in the hPAEpiC membrane, allowing excessive Ca^2+^ influx, and resulting in endoplasmic reticulum (ER) stress [11]. ER stress transfers Ca^2+^ from the ER into mitochondria, disrupting mitochondrial membrane polarity and increasing mitochondrial reactive oxygen species (ROS) production, ultimately promoting NLRP3 inflammasome activation [51,52]. Once activated, NLRP3 can indirectly promote the activation of urokinase plasminogen activator (uPA) and its receptor (uPAR) through proinflammatory cytokines such as IL-1β [53,54]. In this scenario, IL1β plays a central role in barrier disruption. Additionally, NLRP3 activation also facilitates extracellular vesicle (EV) release, propagating proinflammatory and proteolytic signals to neighboring cells [55].

In our current study, we observed that P2X7R activation by ETH, ALD, and e-Cig vapors increased NLRP3 inflammasome activation in hPAEpiCs, promoting the expression and release of uPA from these cells (Figure 1). Notably, our study demonstrates that exposure to e-Cig vape with 0% nicotine significantly increases uPA packaging into lung-derived EVs. It is well established that e-liquid aerosols contain reactive nitrogen species such as nitric oxide (NO) and aldehydes can enhance proinflammatory signaling by increasing IL-1β, TNF-α, and IL-8 levels in lung epithelial cells [56], and can influence uPA expression and release [57]. During NLRP3 inflammasome activation, caspase-1 functions as a core component of the canonical inflammasome complex and is essential for the proteolytic maturation of pro-IL-1β [58]. Activated NLRP3 can also recruit caspase-8, which contributes to IL-1β maturation through non-canonical pathways [59]. The significant upregulation of caspase-1 and caspase-8 expression in hPAEpiCs following ETH, ALD, and e-Cig stimulation supports their coordinated involvement in IL-1β activation and maturation and is associated with increased uPA expression in hPAEpiCs (Figure 2).

Inhalation of nicotine and other abusive drugs not only causes pulmonary fibrosis, but they can also accelerate epithelial-to-mesenchymal transition, worsening the progression of fibrosis [60,61]. In pulmonary fibrosis, uPA plays a protective role by binding to its receptor uPAR and converts plasminogen (an inactive zymogen) into the active protease plasmin, which promotes fibrin degradation. This process helps maintain alveolar structure and restore efficient gas exchange. Activated plasmin is released from lung tissues primarily through the ECM degradation mediated by plasmin itself [62]. Other than lungs, the plasminogen activator system also affects the brain and liver. For example, tissue plasminogen activator (tPA) regulates nicotine-induced reward and dopamine release in the mouse brain [63], while excessive alcohol consumption promotes uPA/tPA-mediated liver damage in the mice [64]. In line with these previous observations, against ETH, ALD, and e-Cig stimulation, hPAEpiCs significantly upregulated extracellular release of uPA via EVs.

uPA can be transported via EVs, and growing evidence indicates that both cancer and immune cells utilize uPA for intercellular communication and regulation of proteolytic activity. Human HT1080 fibrosarcoma cells have been shown to secrete extracellular uPA bound to its receptor uPAR on EVs, suggesting that EV-assisted uPA-uPAR complexes contribute to tumor invasiveness [65]. Likewise, immune cells such as dendritic cells and CD8^+^ T lymphocytes generate exosomes enriched with uPAR signaling components, highlighting their relevance in immune modulation [66]. These findings collectively support the concept that EV-associated uPA and uPAR serve as dynamic biomarkers reflecting both pathological and immune-related cellular activities. In our current study, we reported a significant increase in uPA packaged into hPAEpiC-EVs in response to ETH, ALD, and e-Cig stimulation (Figure 1). Due to their large surface area, EVs offer increased opportunities for uPA-uPAR interactions on recipient cells [65].

In addition to uPA levels, parallel expression of its receptor uPAR is essential for effective plasmin activation. For example, significant upregulation of uPAR expression in axonal injury increased the uPA-uPAR interaction, leading to axonal regeneration [67]. Since uPAR lacks a cytoplasmic domain, it requires association with other transmembrane proteins for intracellular signals. The uPA-uPAR signaling primarily relies on integrins and GPCR responses [56]. Activated integrins initiate downstream ERK/MAPK signaling, a key pathway that can promote cellular uPA expression and release through a positive feedback mechanism [68]. ERK/MAPK signaling can also contribute to activation of matrix metalloproteinases (MMPs) crucial for cell migration and tissue remodeling [69].

Matrix metalloproteinases (MMPs) are proteolytic enzymes that degrade the cellular ECM, facilitate tissue remodeling and cell migration [70]. In addition, MMPs also participate in various pathophysiological processes, including wound healing and neuroinflammation. During cerebral ischemia–reperfusion injury, MMP-2 and MMP-9 activation reduces the tight junction (TJ) protein expression in the brain endothelium, easing the BBB permeability [71]. Similarly, drugs of abuse influence the expression of MMPs in the BBB. Psychostimulants like methamphetamines have been shown to have significant effects on the MMP-2 and MMP-9 activation, resulting in the leakage of BBB [72]. Our earlier work also demonstrated that binge drinking increases the activation of MMP-1 and -9 in hBMVECs, resulting in neuroinflammation [73]. More recently, our in vivo studies also confirmed that ETH inhalation increases the proinflammatory cytokine expression within the BBB, indicative of barrier disruption in mice [74].

In the present study, in vivo results demonstrated that mice exposed to chronic intermittent ethanol (CIE) exposure induced a significant increase in uPA and uPAR gene expression in the brain microvessels (Figure 3A,B) and simultaneously reduced the expression of SERPINE1 (Figure 3E), a major inhibitor of the uPA-uPAR complex [75]. Under these conditions, enhanced uPA-uPAR interaction leads to increased plasmin activation, which can compromise BBB integrity and promote barrier leakage [76]. Along the same lines, CIE exposure also upregulated the MMP-2 and MMP-9 gene expression (Figure 3C,D), while downregulating TIMP1, confirming the potential barrier compromise (Figure 3F). In the context of BBB compromise, imbalance between MMPs and TIMP-1 expression leads to ECM modification, resulting in scarring and fibrosis [77]. The increased expression of the Col-1α1 gene in mouse brain microvessels (Figure 3G) also supports ethanol-induced fibrotic remodeling and repair of the BBB [78].

Alcohol-induced cerebral microvessel damage was further validated by assessing the presence of total IgG in the brain parenchyma. Under physiological conditions, total IgG does not cross the BBB due to its large molecular size; therefore, its detection within the brain is widely accepted as an indicator of BBB disruption and vascular leakage [79]. Consistent with ECM remodeling-gene expression changes, brain lysates from CIE mice showed fractions of total IgG, further supporting alcohol-induced vascular compromise (Figure S1). In addition, elevated levels of IgG heavy- and light-chain fragments in CIE brain lysates further validate BBB dysfunction.

Notably, IgG light-chain signals were detected across the control group as well. Given their smaller molecular size (~25 kDa), free light chains might show limited passage across biological barriers, and low basal levels have been reported in the brain under physiological conditions [80,81]. However, the marked increase observed in CIE brains suggests enhanced permeability and increased immunoglobulin extravasation following alcohol exposure (Figure S1).

Repair of the BBB is a complex process with both protective and compromising effects, and the plasminogen activator system (uPA-uPAR) plays a multifaceted role in the homeostasis of BBB integrity [82]. While fibroblasts produce factors that help repair the BBB after injury [78], the pathophysiological activation of plasmin by uPA-uPAR interaction can lead to a breakdown of the fibrin barrier, inhibiting proper healing [83]. For instance, mice exposed to peripheral thermal injury exhibited elevated uPA expression in the brain, resulting in enhanced BBB compromise [84]. In lung endothelial cells, nitric oxide treatment increases uPA levels, disrupting the critical cell–cell interactions necessary for vascular integrity [85].

Next, we investigated the effect of uPA on compromised barrier integrity by supplementing the hBMVEC monolayer with human fibrinogen (Fg, 0.5 mg/mL) to mimic a fibrotic microenvironment. TEER values were measured continuously to assess changes in barrier integrity.

Upon fibrinogen addition, brain endothelial cells are known to promote thrombin generation, a key enzyme that converts fibrinogen into an insoluble fibrin network [86,87]. In our experiments, supplementation of fibrinogen to the hBMVEC monolayer increased TEER values, consistent with the formation of a fibrin-rich scaffold that transiently enhances barrier resistance. Subsequent stimulation with hPAEpiC-EVs carrying uPA cargo led to a significant drop in TEER values, reflecting fibrinolysis (Figure 4C). As a control, a parallel TEER study without fibrinogen treatment was performed and showed a modest drop in electrical resistance as compared to the test groups, supporting the idea that significant TEER reduction was primarily due to fibrinolysis (Figure 4A).

In this study, the reduction in TEER reflects compromise of the brain endothelial barrier mediated by ECM remodeling and degradation. Increased uPA activity is known to enhance plasminogen activation, leading to plasmin generation, and resulting in ECM remodeling [88]. Consistent with this mechanism, TEER measurements showed that EVs isolated from BC11-pretreated hPAEpiCs protected the barrier compromise in hBMVECs, supporting a potential role of uPA-containing EVs in BBB-associated ECM remodeling (Figure 5). During ECM remodeling, plasmin initiates MMP-9 activity, and together these two proteases synergistically accelerate ECM degradation [89]. MMP-9 activation by plasmin occurs through both direct and indirect mechanisms. Directly, uPA can cleave and activate pro-MMP-9 at both of its N- and C-termini [90]. Alternatively, uPA activates plasmin, which then activates MMP-9 [91], leading to the subsequent activation of latent TGF-β1 within cellular ECM [92]. This dual mechanism highlights a significant link between uPA and MMP-9 in regulating ECM remodeling. Additionally, TGF-β1 further promotes ECM degradation by suppressing the activity of matrix-stabilizing factors such as tissue inhibitors of metalloproteinases (TIMPs).

In our study, stimulation of hBMVECs with hPAEpiC-EVs generated after ETH, ALD, and e-Cig exposure significantly increased levels of the plasmin (50–100 pg/mL) and active MMP-9 (0.5–2 ng/mL). In contrast, EVs from P2X7R antagonist pretreated cells loaded with lower uPA cargo markedly lowered the plasmin conversion (Figure 6A) and MMP-9 activity (Figure 6B). Consistent with this, higher active TGF-β1 expression was observed in hBMVEC lysates treated with EVs (Figure 6D), indicating activation of the uPA–MMP-9–TGF-β1 axis and enhanced ECM degradation. hPAEpiC pretreatment with the uPA inhibitor, BC11, significantly reduced EV-uPA cargo after exposure to ETH, ALD and e-Cig (Figure 5A). Stimulation of hBMVECs with these EVs resulted in attenuated extracellular release of active MMP-9 and the maturation of TGF-β1 (Figure S2), thereby supporting a mechanistic role for uPA in the uPA–MMP9–TGF-β1 axis. Of note, treatment with the P2X7R antagonist failed to reduce levels of activated TGF-β1 following e-Cig exposure (1.8% nicotine), suggesting the involvement of alternative pathways. One possible mechanism is the direct activation of TGF-β1 by uPA through cleavage of the latency-associated peptide from the latent TGF-β1 complex [93], bypassing MMP-9-mediated activation.

P2X7R plays a key role in ETH, ALD and e-Cig effects since hPAEpiCs pretreated with P2X7R inhibitor decreased the NLRP3 inflammasome activation and reduced IL-1β secretion. Similarly, mice treated with a P2X7R inhibitor demonstrated normalization of expression of all dysregulated BBB compromising genes. Further, EVs derived from hPAEpiC pretreated with the P2X7R antagonist had much attenuated active MMP-9 release and TGF-β1 expression in brain endothelial cells when compared to effects of EVs without P2X7R inhibition. All these results indicate a key role of P2X7R signaling in mediating changes caused by ETH, ALD or e-Cig (Schematic explains the sequence of molecular events in this work).

## 5. Conclusions

Collectively, these data support a model in which ETH, ALD, and e-Cig vapor exposure activate P2X7R in hPAEpiCs, triggering NLRP3 inflammasome activation and release of uPA-enriched EVs. These EVs engage uPAR on brain endothelial cells, enhancing plasminogen-to-plasmin conversion, activating MMP-9, and subsequently promoting latent TGF-β1 maturation. The resulting proteolytic ECM remodeling compromises BBB integrity. Pharmacological inhibition of P2X7R mitigates this cascade, highlighting its potential as a therapeutic target to preserve BBB function in the context of alcohol misuse and e-Cig exposure.

## Figures and Tables

**Figure 1:**
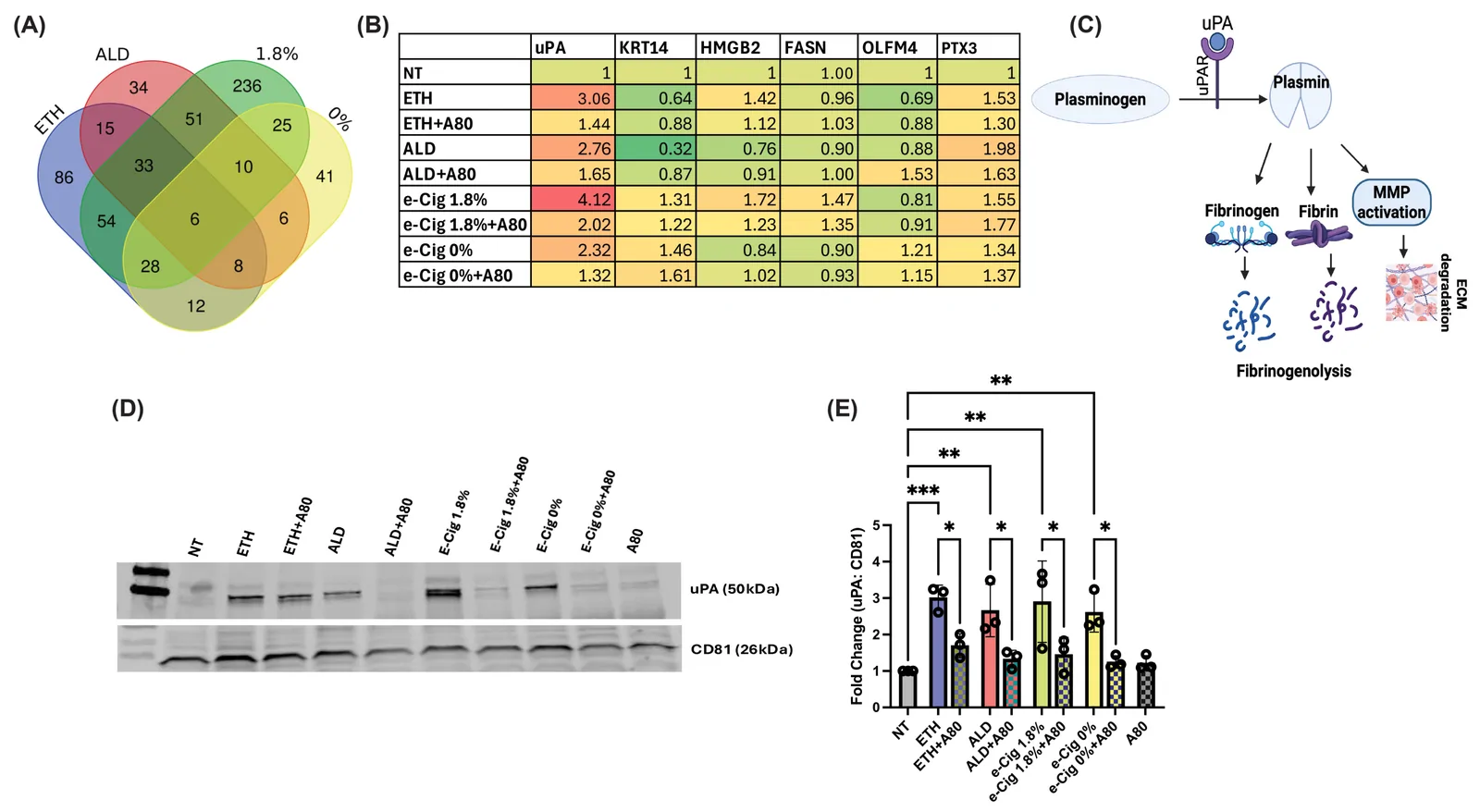
**ETH, ALD, and e-Cig exposure enhance uPA cargo in hPAEpiC-derived EVs.** Proteins isolated from hPAEpiC-derived EVs were analyzed using LC-MS/MS with a data-independent acquisition (DIA) workflow. (**A**) Venn diagram showing six proteins commonly upregulated across treatments. uPA: urokinase plasminogen activator; KRT4: keratin 14; HMGB2: High-Mobility Group Box 2; FASN: fatty acid synthase; OLFM4: Olfactomedin 4; PTX3: Pentraxin 3. (**B**) Heat map of EV protein expression, normalized to non-treated lung EVs, indicating fold changes relative to control. (**C**) Schematic of uPA-mediated proteolysis, showing fibrin/fibrinogen degradation and subsequent ECM breakdown. (**D**) Representative Western blot demonstrating elevated uPA levels in hPAEpiC-EVs following exposure. (**E**) Densitometric quantification of EV-uPA protein expression from three independent experiments. Statistical analysis: one-way ANOVA; * *p* ≤ 0.05, ** *p* ≤ 0.01, *** *p* ≤ 0.001.

**Figure 2:**
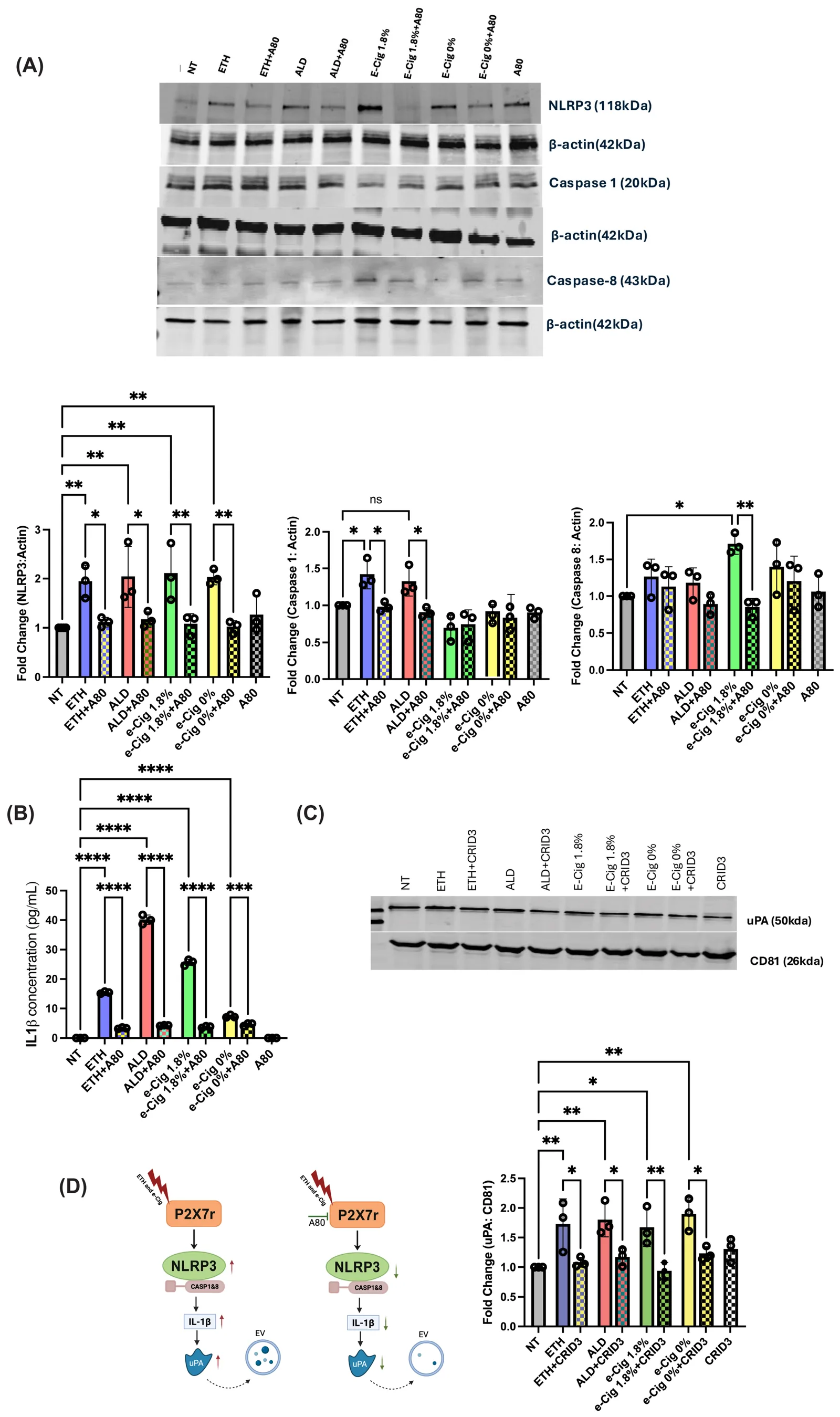
**P2X7 receptor activation by ETH, ALD, and e-Cig triggers NLRP3 inflammasome-dependent uPA expression in hPAEpiCs.** (**A**) Western blots of hPAEpiC lysates probed for NLRP3, Caspase-1, and Caspase-8. Bar graphs indicate fold changes relative to non-treated controls (NTs). Overnight exposure increased NLRP3 and Caspase-1 expression ~2-fold. Pretreatment with P2X7R antagonist A80 attenuated these effects. (**B**) Mature IL-1β levels in culture supernatants measured by ELISA. (**C**) Immunoblot of hPAEpiC-derived EV lysates showing reduced uPA loading upon CRID3-mediated NLRP3 inhibition; CD81 serves as loading control. (**D**) Schematic illustrating P2X7R-driven signaling cascade leading to uPA-containing EV release. Statistical analysis: one-way ANOVA; * *p* ≤ 0.05, ** *p* ≤ 0.01, *** *p* ≤ 0.001, **** *p* ≤ 0.0001, ns = non-significant.

**Figure 3:**
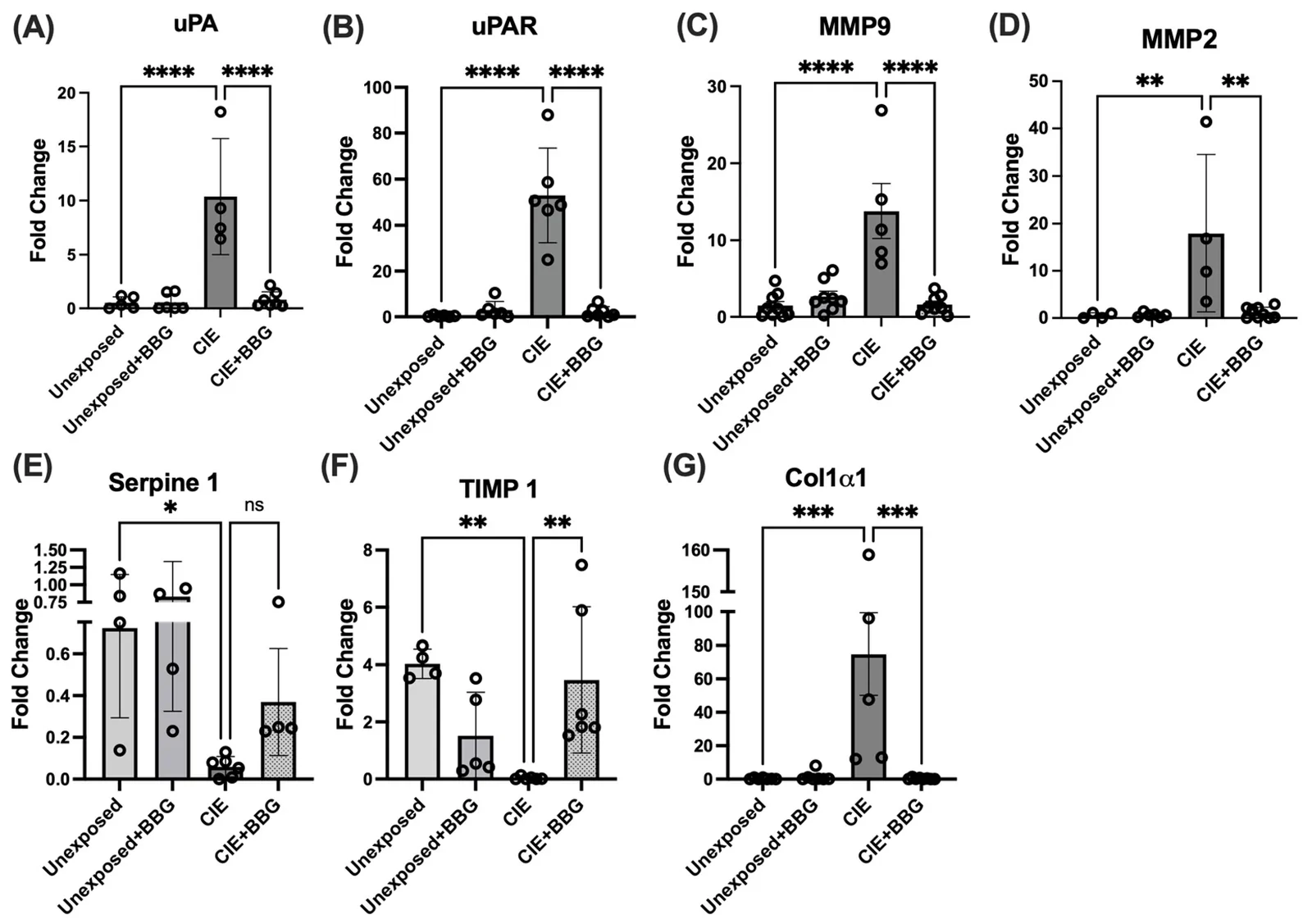
**Alcohol-induced P2X7R activation upregulates ECM-degrading genes in mouse brain microvessels, indicating BBB compromise in vivo.** (**A**–**D**) qPCR analysis of microvessels from mice exposed to chronic ethanol (ETH) vapor shows 20–100-fold increases in uPA, uPAR, MMP-2, and MMP-9 expression. (**E**,**F**) ECM-protective genes (serpine-1, TIMP1) were significantly downregulated. (**G**) Col1α1 expression, a wound-healing marker, confirms BBB compromise. Pretreatment with P2X7R antagonist BBG normalized gene expression and reduced BBB injury. Statistical analysis: one-way ANOVA; * *p* ≤ 0.05, ** *p* ≤ 0.01, *** *p* ≤ 0.001, **** *p* ≤ 0.0001, ns = not significant.

**Figure 4:**
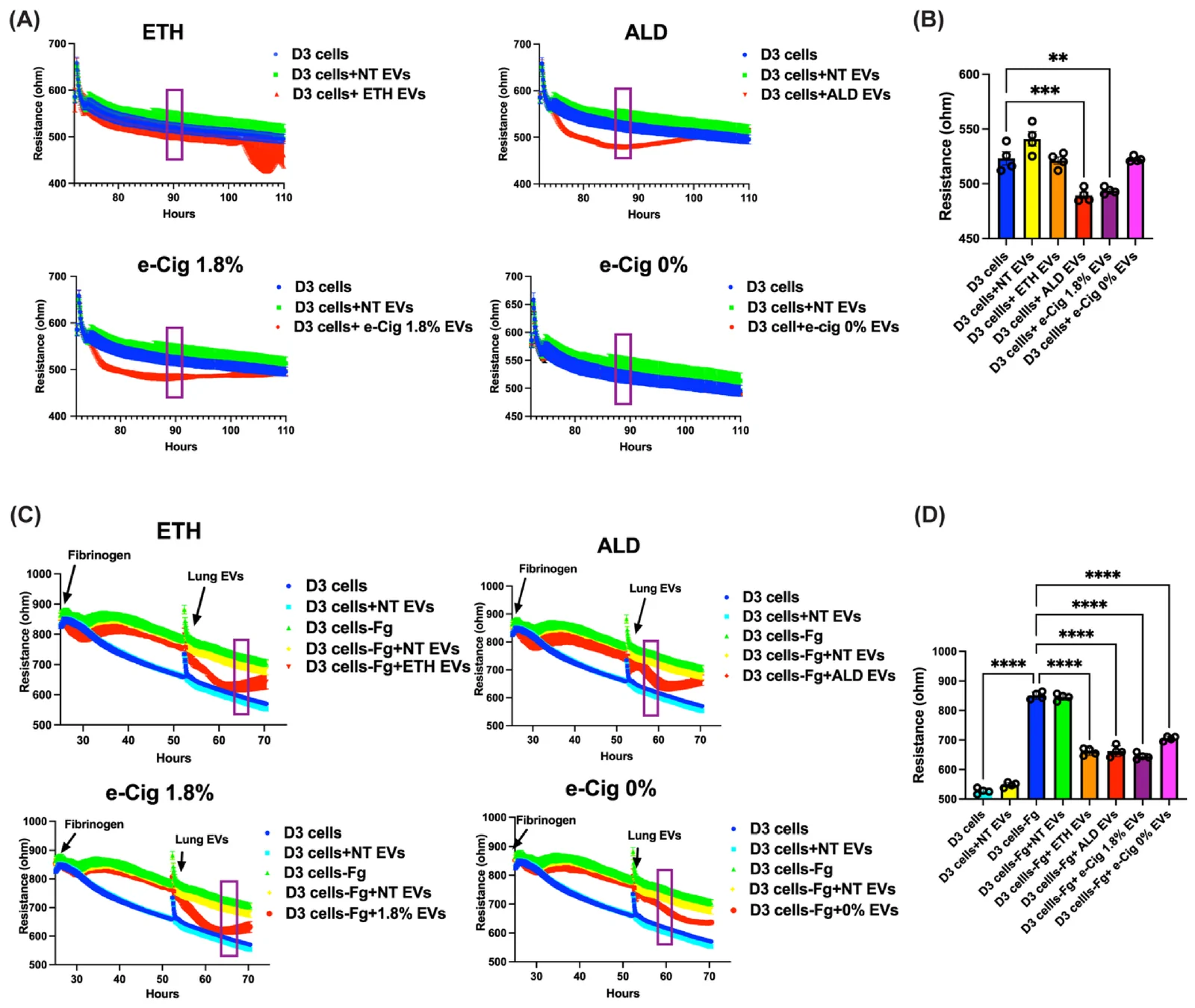
**hPAEpiC-derived EVs reduce trans-endothelial electrical resistance (TEER) in hBMVECs, indicating endothelial barrier disruption**. hBMVECs were grown on TEER plates until stable baseline values were achieved. Cells were pretreated with or without native human fibrinogen and TEER values recorded continuously at 4000 Hz. EV stimulation (1:500) was applied for ≥24 h. (**A**) Without fibrinogen, hBMVECs exhibited moderate TEER reduction following exposure to ALD and 1.8% nicotine-derived EVs. (**B**) Control TEER measurements for statistical comparison. (**C**,**D**) Fibrinogen pretreatment amplified TEER reduction (20–30%) in response to ETH, ALD, or e-Cig EVs. EVs from A80-pretreated cells did not affect TEER. Arrows indicate stimulation time points; boxed region denotes analysis window. Statistical summary: one-way ANOVA; ** *p* ≤ 0.01, *** *p* ≤ 0.001, **** *p* ≤ 0.0001.

**Figure 5:**
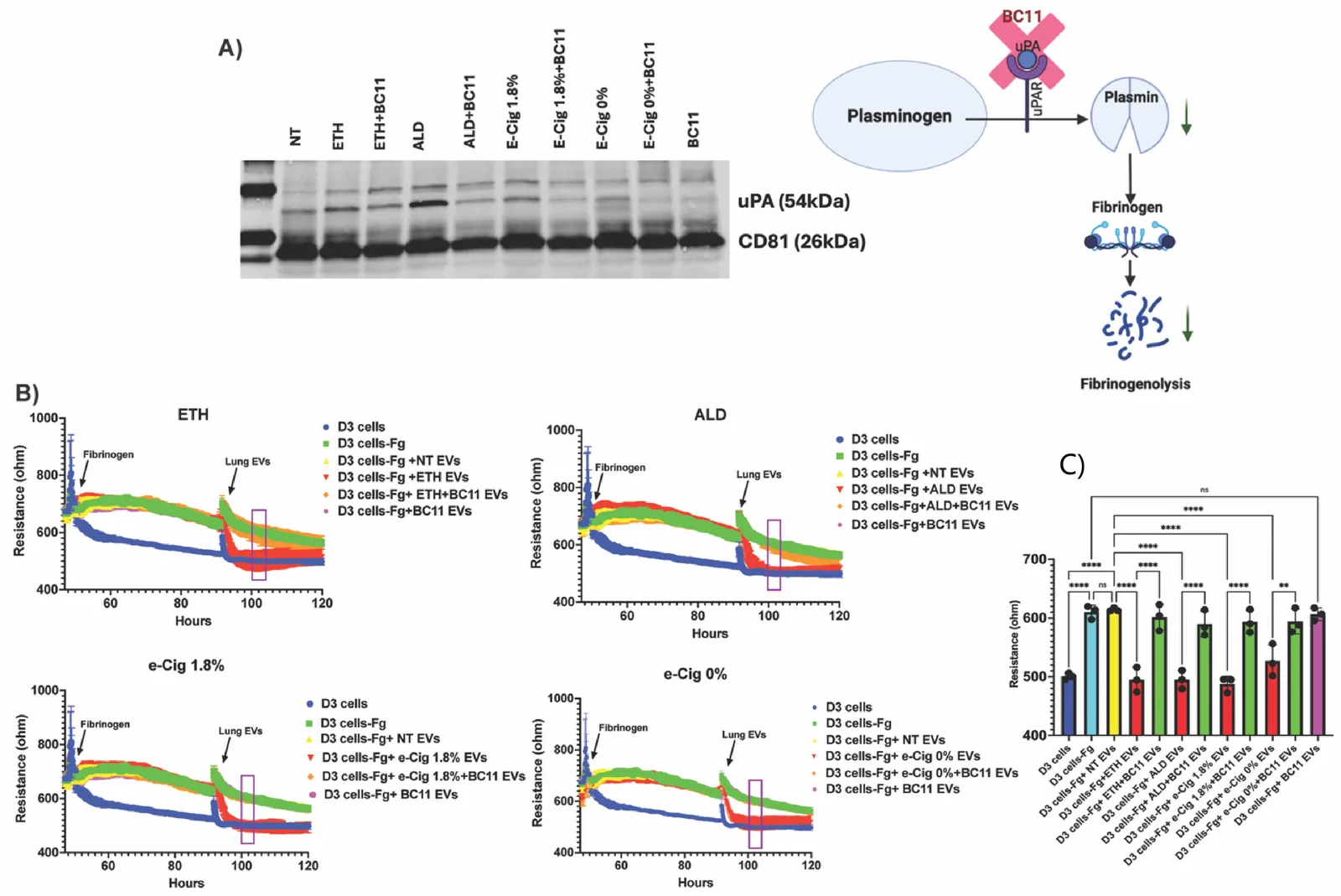
**uPA-enriched hPAEpiC-EVs mediate ECM degradation and compromise BBB integrity.** hPAEpiCs were pretreated with uPA inhibitor BC11 for 3 h prior to ETH, ALD, or e-Cig exposure. Isolated EVs were applied to hBMVECs in TEER assays. (**A**) Western blot confirms BC11 effectively suppresses uPA loading into EVs. (**B**) uPA-enriched EVs induced a 10–20% reduction in TEER, whereas EVs from BC11-pretreated cells did not alter TEER. Arrows indicate fibrinogen (Fg) and EV stimulation time points; boxed region denotes analysis window. (**C**) Bar graphs summarize the statistical comparison. One-way ANOVA; ** *p* ≤ 0.01, **** *p* ≤ 0.0001, ns = non-significant.

**Figure 6:**
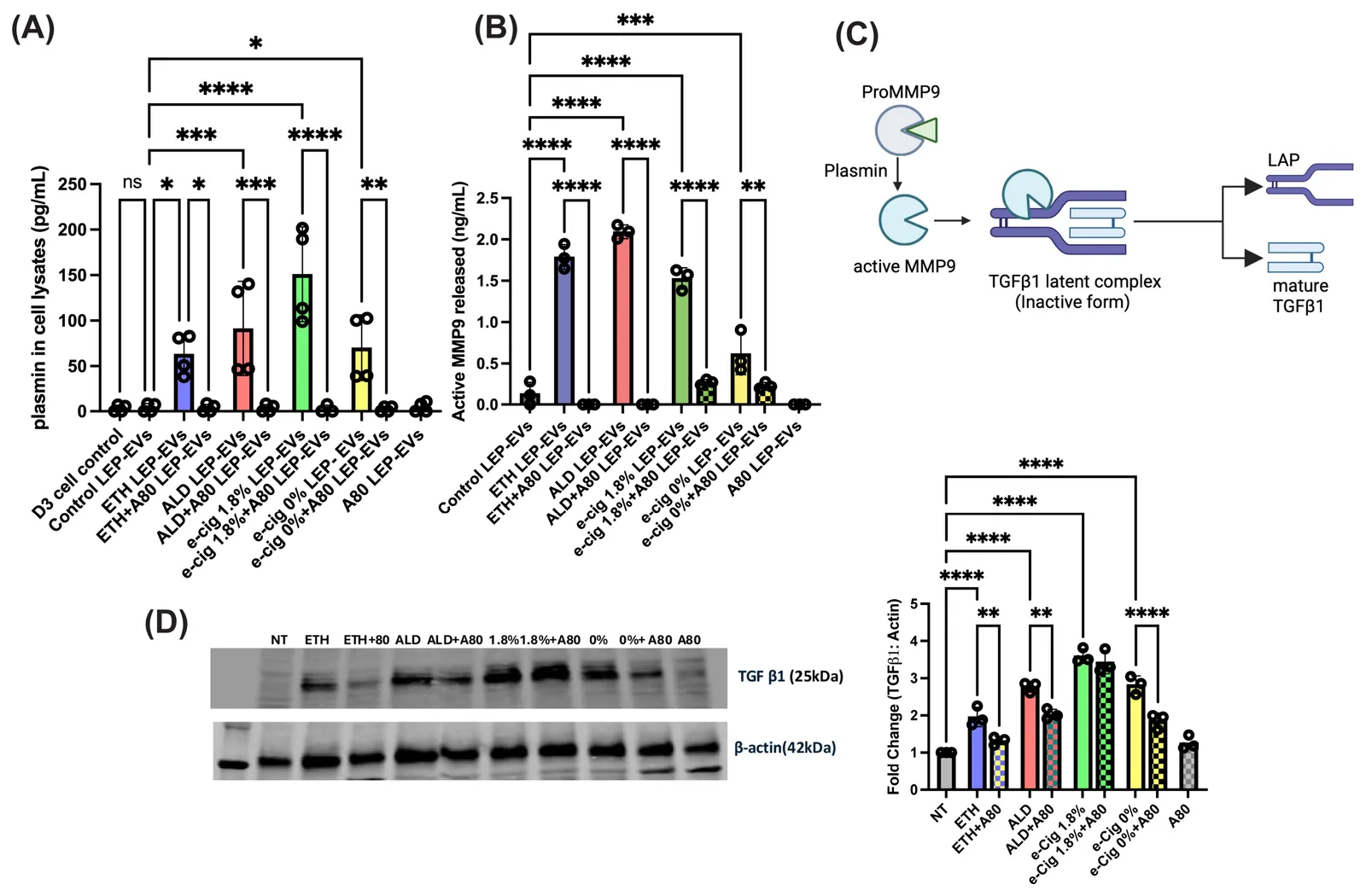
**uPA-containing hPAEpiC-EVs activate plasmin, MMP-9, and TGF-β1 in hBMVECs, promoting ECM degradation.** Confluent hBMVECs were stimulated overnight with EVs (1:500). Lysates and supernatants were analyzed by ELISA. (**A**) EVs from ETH-, ALD-, and e-Cig-conditioned media increased lysate plasmin levels up to 200 pg/mL. (**B**) Active MMP-9 secretion increased by ~1.2 ng/mL. (**C**,**D**) Activated MMP-9 cleaves latent TGF-β1, promoting TGF-β1 maturation. EVs from A80-pretreated cells showed reduced plasmin, MMP-9, and TGF-β1 activation, except in 1.8% nicotine group, suggesting alternative pathways. Statistical analysis: one-way ANOVA; * *p* ≤ 0.05, ** *p* ≤ 0.01, *** *p* ≤ 0.001, **** *p* ≤ 0.0001.

## Data Availability

The original contributions presented in this study are included in the article/Supplementary Material. Further inquiries can be directed to the corresponding authors.

## Supplementary Materials

The following supporting information can be downloaded at https://www.mdpi.com/article/10.3390/cells15191750/s1, Figure S1: Alcohol-induced P2X7R activation increases IgG leakage into the mouse brain, indicating blood–brain barrier (BBB) compromise in vivo. (A) Western blot analysis of total mouse brain lysates showed no detectable intact IgG levels in unexposed control mouse brains, whereas alcohol-exposed (CIE) brains showed significantly higher IgG levels. Similarly, accumulation of IgG heavy and light chains was significantly elevated in CIE mice. Pre-treatment with the P2X7R antagonist BBG significantly reduced IgG leakage into the brain parenchyma, indicating its potential BBB-protective role. (B) Bar graphs summarize the statistical comparison. One-way ANOVA; * *p* ≤ 0.05, ** *p* ≤ 0.01, *** *p* ≤ 0.001, **** *p* ≤ 0.0001. Figure S2: Inhibition of ETH- and e-cigarette-induced uPA expression in hPAEpiCs by BC11 attenuates MMP9 activation and TGF-β1 expression in hBMVECs. (A) EVs derived from hPAEpiCs exposed to ETH, ALD, and e-cigarette vapor (1.8% nicotine) significantly increased levels of active MMP9, and (B) TGF-β1 expression in hBMVECs. In contrast, EVs isolated from BC11-pretreated hPAEpiCs markedly reduced MMP9 activation and TGF-β1 expression in hBMVECs. (C) Bar graphs represent densitometric quantification of Western blot data for statistical analysis. One-way ANOVA; * *p* ≤ 0.05, ** *p* ≤ 0.01, *** *p* ≤ 0.001, **** *p* ≤ 0.0001. Table S1: hPAEpiC-extracellular vesicle (EV) particle size and numbers against drugs of abuse.

## Abbreviations

P2X7R	Purinergic receptor P2X7
uPA	Urokinase-type plasminogen activator
uPAR	Urokinase plasminogen activator surface receptor
hPAEpiCs	Human pulmonary alveolar epithelial cells
hBMVECs	Human brain microvascular endothelial cells
TEER	Transendothelial electrical resistance
EVs	Extracellular vesicles
BBB	Blood–brain barrier
CIE	Chronic intermittent ethanol (CIE)
BBG	Brilliant Blue G
NLRP3	Nod-like receptor pyrin domain containing 3
mtDNA	Mitochondrial DNA
TGF-Β1-1β	Transforming growth factor beta 1
IL-1β	Interleukin 1 beta
MMP	Matrix metalloproteinase
TIMP1	Tissue inhibitor of metalloproteinase 1
