# Supplementary material for: Alcohol and E-Cigarettes Activate P2X7R in Lung Epithelial Cells, Stimulating Release of Plasminogen Activator in Extracellular Vesicles, Injuring Blood-Brain Barrier
Source: Cells. 2026 Sep 25;15(19):1750. doi: 10.3390/cells15191750 (PMC13664298; doi:10.3390/cells15191750)
Supplement: Supplementary file 1 [file cells-15-01750-s001.zip › cells-4574727-supplementary.pdf]

(A)

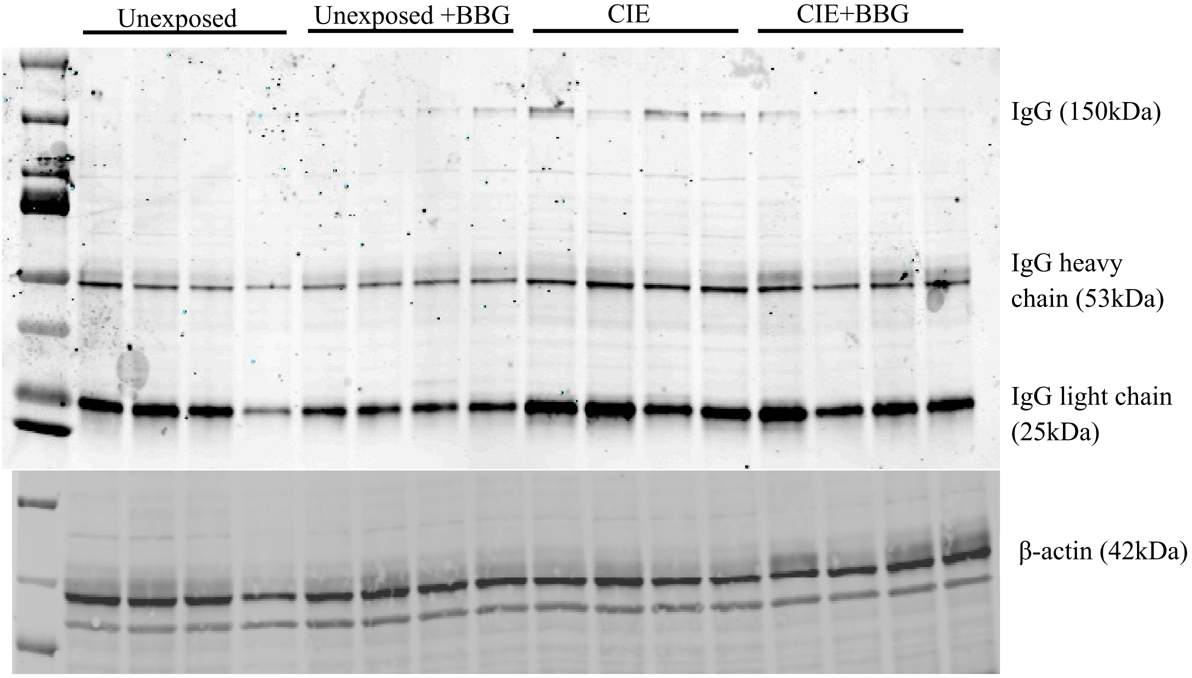

(B)

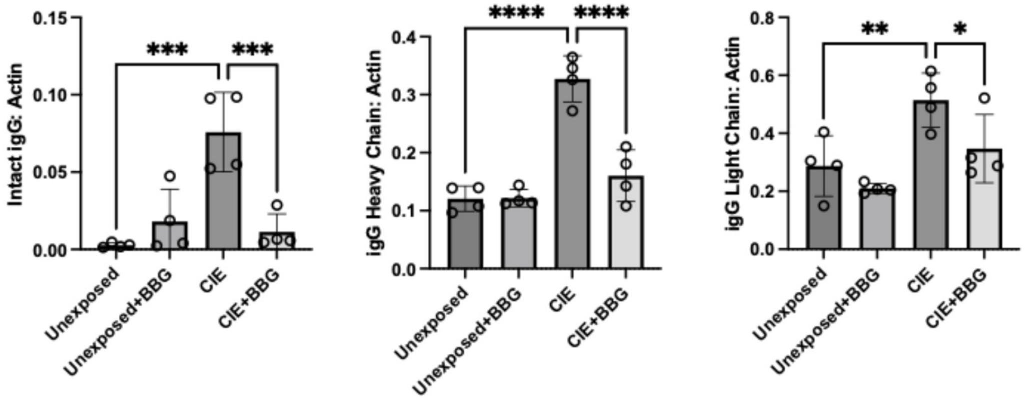

Figure S1: Alcohol-induced P2X7R activation increases IgG leakage into the mouse brain, indicating blood–brain barrier (BBB) compromise in vivo. (A) Western blot analysis of total mouse brain lysates showed no detectable intact IgG levels in unexposed control mouse brains, whereas alcohol-exposed (CIE) brains showed significantly higher IgG levels. Similarly, accumulation of IgG heavy and light chains was significantly elevated in CIE mice. Pre-treatment with the P2X7R antagonist BBG significantly reduced IgG leakage into the brain parenchyma,

indicating its potential BBB-protective role. **(B)** Bar graphs summarize the statistical comparison. One-way ANOVA; $*p \leq 0.05$, $**p \leq 0.01$, $***p \leq 0.001$, $****p \leq 0.0001$.

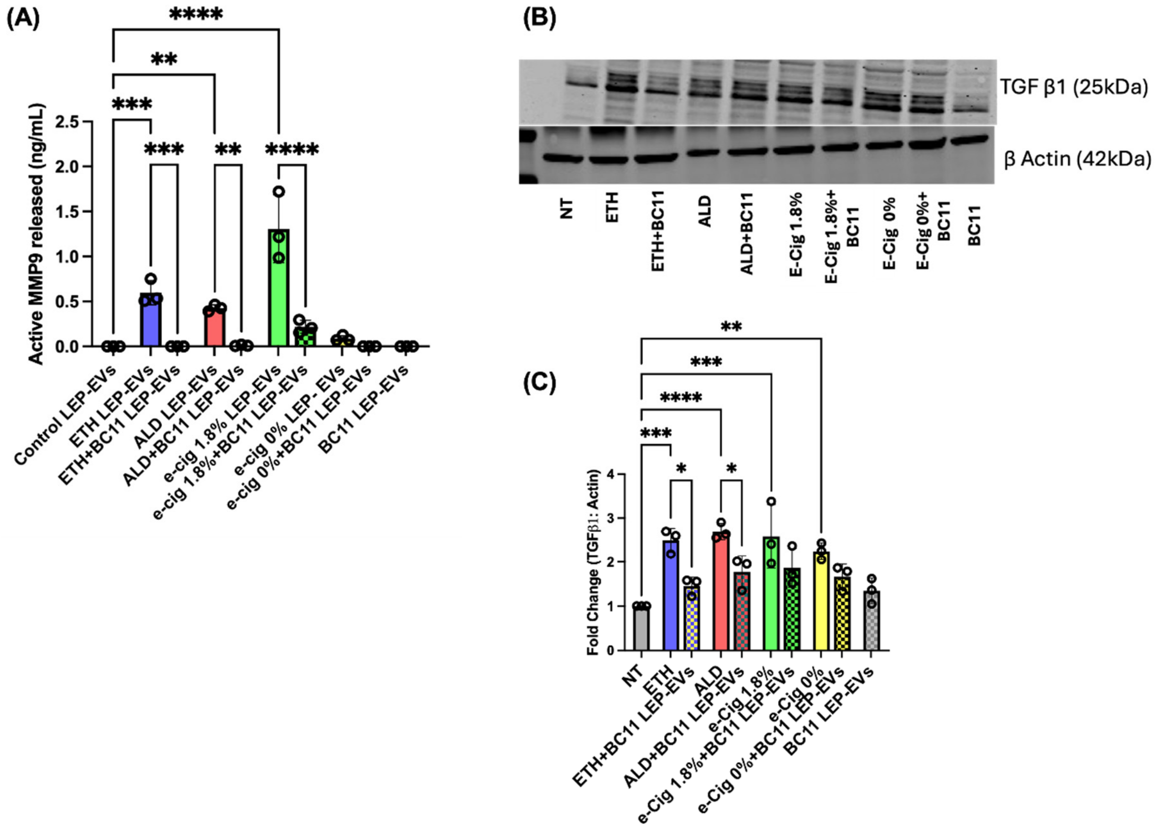

Figure S2: Inhibition of ETH- and e-cigarette–induced uPA expression in hPAEpiCs by BC11 attenuates MMP9 activation and TGF-β1 expression in hBMVECs. (A) EVs derived from hPAEpiCs exposed to ETH, ALD, and e-cigarette vapor (1.8% nicotine) significantly increased levels of active MMP9, and (B) TGF-β1 expression in hBMVECs. In contrast, EVs isolated from BC11-pretreated hPAEpiCs markedly reduced MMP9 activation and TGF-β1 expression in hBMVECs. (C) Bar graphs represent densitometric quantification of Western blot data for statistical analysis. One-way ANOVA; $*p \leq 0.05$, $**p \leq 0.01$, $***p \leq 0.001$, $****p \leq 0.0001$.

Table S1: hPAEpiC-extracellular vesicle (EV) particle size and numbers against drugs of abuse.

S.No	Treatment	Mean +/- standard error	Mode +/- standard error	Concentration (particles/mL)
1	Nontreated	*257.4 +/- 42.0 nm*	*226.5 +/- 64.6 nm*	*2.56e+07*
2	ETH	*307.1 +/- 22.6 nm*	*343.7 +/- 42.3 nm*	*1.73e+08*
3	ETH+A80	*298.1 +/- 47.9 nm*	*203.4 +/- 56.4 nm*	*7.81e+07*
4	ALD	*306.8 +/- 50.2 nm*	*301.8+/- 38.3 nm*	*3.61e+07*
5	ALD+A80	*283.4 +/- 51.5 nm*	*241.0 +/- 48.3 nm*	*3.18e+07*
6	e-Cig 1.8%	*332.9 +/- 39.6 nm*	*341.7 +/- 48.8 nm*	*5.90e+07*
7	e-Cig 1.8%+A80	*214.4 +/- 51.9 nm*	*144.8 +/- 52.1 nm*	*3.17e+07*
8	e-Cig 0%	*390.0 +/- 33.0 nm*	*299.9 +/- 73.5 nm*	*3.33e+07*
9	e-Cig 0%+A80	*272.1 +/- 51.6 nm*	*248.6 +/- 115.1 nm*	*6.31e+07*
10	A80	*267.5 +/- 38.9 nm*	*232.9 +/- 49.5 nm*	*2.19e+07*
